# Supplementary material for: Starvation transforms signal encoding in C. elegans thermoresponsive neurons and suppresses heat avoidance via bidirectional glutamatergic and peptidergic signaling
Source: eLife. 2026 Oct 9;14:RP108246. doi: 10.7554/eLife.108246 (PMC13652537; doi:10.7554/eLife.108246)
Supplement: Peer review history

# Editor report: eLife Assessment

**DOI:** 10.7554/eLife.108246.3.sa0

**Authors:** Sonia Q Sen

**Affiliations:** Tata Institute for Genetics and Society India

**Keywords:** Convincing, Important

This **important** study shows how hunger alters avoidance of harmful heat in *C. elegans* by reconfiguring the activity of key sensory neurons. The evidence is **convincing**, with well-designed behavioural, genetic, and imaging experiments that support the main conclusions. The work will be of interest to neuroscientists studying how internal states shape sensory processing and behaviour across species.

# Referee report: Reviewer #1 (Public review)

**DOI:** 10.7554/eLife.108246.3.sa1

This study by Thapliyal and Glauser investigates the neural mechanisms that contribute to the progressive suppression of thermonociceptive behavior that is induced under conditions of starvation. Several previous studies have demonstrated that when starved, *C. elegans* alters its preferences for a variety of sensory cues, including CO2, temperature, and odors, in order to prioritize food seeking over other behavioral drives. The varied mechanisms that underlie the ability of internal states to alter behavioral responses are not fully understood, however there is growing evidence for a role by neuropeptidergic signaling as well as capacity for functionally distinct microcircuits, formed by distinct internal states, to trigger similar behavior outcomes.

Within the physiological range of *C. elegans* (~15-25C), starvation triggers a profound reduction in temperature-driven thermotaxis behaviors. This reduction involves the recruitment of the amphid sensory neuron pair AWC. The AWC neurons primarily act to sense appetitive chemosensory cues, however under starvation conditions begin to display temperature responses that previous studies have linked to the reduction in thermotaxis navigation. Here, Thapliyal and Glauser investigate the impact of starvation on thermonociceptive responses, innate escape behaviors that are triggered by exposure to noxious temperatures above 26C or rapid thermal stimuli below 26C. They compare the strength of thermonociceptive behaviors, specifically heat-triggered reversals, in worms experiencing either early food deprivation (1 hour off food) or prolonged starvation (6 hours off food). Their experiments demonstrate a progressive loss of heat-triggered reversals that is mediated by AWC and ASI neurons, as well as both glutamateric and neuropeptidergic signaling.

At the level of neural activity, this study reports that the transition from early food deprivation to prolonged starvation reconfigures the temperature-driven activity of AWC neurons from mostly excitatory to a heterogenous mix combining excitatory and inhibitory responses. This finding is interesting in light of previous work that reported the opposite transition in temperature-driven AWC responses when comparing well-fed worms to those kept from food for 3 hours. Specifically, these differences highlight the differences between temperature responses within the *C. elegans* physiological temperature range (previous studies) and their noxious temperature response (this study). This study also identifies neural and genetic mechanisms that contribute to differences in thermonociceptive responses at +1 versus +6 hours starvation; interestingly, these mechanisms are also partially distinct from those that contribute to differences in negative thermotaxis behaviors in well-fed and +3 hours starvation worms. A limitation of this manuscript is that these differences are not particularly acknowledged or addressed, other than the hypothesis that independent mechanisms underlie negative thermotaxis versus thermonociceptive stimuli.

In this revised article, the authors commanding knowledge of the distinction between thermotaxis navigation (especially negative thermotaxis) and thermonociceptive behaviors is communicated with an admirable depth and clarity to the readers; this study's new findings are helpfully contextualized within the previous literature.

This study represents an important addition to the growing evidence that *C. elegans* sensory behaviors are strongly impacted by internal states, and that neuropeptigergic signaling plays a key role in mediating behavioral plasticity. To that end, the authors have provided compelling evidence of their claims.

# Referee report: Reviewer #3 (Public review)

**DOI:** 10.7554/eLife.108246.3.sa2

Thapliyal, Gopinath, and Glauser show that starvation alters how *C. elegans* respond to noxious thermal stimuli. Using targeted neural ablation, mutant analysis, and live-cell functional imaging the authors demonstrate that hunger changes the properties of AWC sensory neurons, which sense noxious heat. The authors further show that effects of hunger on nociception require ASI neurons, which are known to respond to hunger and mediate effects of food deprivation on behavior. Finally, the study uses mutant analysis to implicate glutamate and specific neuropeptides in thermal nociception and in modulation of nociceptors by hunger-responsive neurons.

The study clearly shows a strong effect of hunger on nociception and documents a striking effect of hunger on the intrinsic properties of AWC sensory neurons, which respond to noxious heat. The study also clearly and compellingly demonstrates that ablation of hunger-responsive ASI neurons blocks effects of hunger on nociceptive AWCs. These data, which constitute the kernel of the manuscript, are striking and exciting. This revised manuscript analyzes effects of starvation on AWC physiology and clearly shows that starvation alters the way AWCs respond to thermal stimuli by decreases the probability that AWCs will be activated and increases the probability that they will be inhibited. New data also identify ASI-derived neuropeptides that are required for modulation of AWCs by starvation. This study reveals a mechanistic link between an animal's metabolic state and sensory processing and establishes modulation of AWC function as a powerful model to study the molecular basis of this link.

# Author comment: Author response

**DOI:** 10.7554/eLife.108246.3.sa3

**Authors:** Saurabh Thapliyal, Parvathi Sushama Gopinath, Dominique A Glauser

**Affiliations:** University of Fribourg Fribourg Switzerland; University of Fribourg Fribourg Switzerland; University of Fribourg Fribourg Switzerland

The following is the authors’ response to the original reviews.

We believe that the manuscript has been substantially strengthened through the revision process. The main changes are summarized below:

We substantially revised the Introduction and Discussion sections to better position our work relative to previous studies on starvation-dependent thermotaxis plasticity, neuropeptidergic modulation, and AWC function.

We clarified throughout the manuscript the distinction between negative thermotaxis in innocuous thermal ranges and thermonociceptive responses to noxious heat. We now discuss more explicitly that these behaviors involve at least partly distinct molecular, cellular, and circuit-level mechanisms.

We performed new experiments in *ins-1* mutants. Unlike what was previously reported for thermotaxis plasticity, *ins-1* does not appear required for starvation-dependent thermonociceptive plasticity in our paradigm (new Figure 6—figure supplement 1).

We revised the analysis and terminology used for AWC calcium imaging data. We no longer use the “deterministic/stochastic” terminology and instead describe a starvation-induced shift from predominantly excitatory responses to a mixed distribution of excitatory and inhibitory responses. We also added new quantitative analyses and histogram representations of response distributions, as directly suggested by reviewers, to better illustrate this point.

We performed new genetic interaction experiments using *eat-4*; *flp-6* double mutants. These analyses revealed that glutamatergic and FLP-6 signaling act largely in parallel to mediate heat-evoked reversals after early food deprivation, while prolonged starvation reveals a hierarchical interaction between these pathways.

We revised and clarified the mechanistic model figures accordingly, particularly regarding the proposed ASI → AWC signaling pathway and the role of ASI-derived neuropeptides.

We improved the presentation and statistical rigor throughout the manuscript, including:

- Replacement of heating power values by corresponding temperature increases,

- Clarification of the rationale for using the 1-hour off-food condition as reference,

- Expanded statistical reporting and multiple-comparison procedures,

- Additional methodological details for calcium imaging, rescue validation, and cell ablation approaches,

- Clarification of replotted datasets in figure legends.

We also simplified the manuscript by removing experiments whose interpretation remained ambiguous (notably the *nsy-1* and *nsy-7* analyses).

Below, we provide a detailed point-by-point response to all reviewer comments.

**Public Reviews:**

**Reviewer #1 (Public review):**

This study by Thapliyal and Glauser investigates the neural mechanisms that contribute to the progressive suppression of thermonociceptive behavior that is induced under conditions of starvation. Several previous studies have demonstrated that when starved, *C. elegans* alters its preferences for a variety of sensory cues, including CO2, temperature, and odors, in order to prioritize food seeking over other behavioral drives. The varied mechanisms that underlie the ability of internal states to alter behavioral responses are not fully understood; however, there is growing evidence for a role of neuropeptidergic signaling as well as the capacity for functionally distinct microcircuits, formed by distinct internal states, to trigger similar behavior outcomes.

Within the physiological range of *C. elegans* (~15-25{degree sign}C), starvation triggers a profound reduction in temperature-driven thermotaxis behaviors. This reduction involves the recruitment of the amphid sensory neuron pair AWC. The AWC neurons primarily act to sense appetitive chemosensory cues; however, under starvation conditions begin to display temperature responses that previous studies have linked to the reduction in thermotaxis navigation. Here, Thapliyal and Glauser investigate the impact of starvation on thermonociceptive responses, innate escape behaviors that are triggered by exposure to noxious temperatures above 26{degree sign}C or rapid thermal stimuli below 26{degree sign}C. They compare the strength of thermonociceptive behaviors, specifically heat-triggered reversals, in worms experiencing either early food deprivation (1 hour off food) or prolonged starvation (6 hours off food). Their experiments demonstrate a progressive loss of heattriggered reversals that is mediated by AWC and ASI neurons, as well as both glutamatergic and neuropeptidergic signaling.

At the level of neural activity, this study reports that the transition from early food deprivation to prolonged starvation reconfigures the temperature-driven activity of AWC neurons from largely deterministic to stochastic. This finding is interesting in light of previous work that reported the opposite transition (from stochastic to deterministic) in temperature-driven AWC responses when comparing well-fed worms to those kept from food for 3 hours. This study also identifies neural and genetic mechanisms that contribute to differences in thermonociceptive responses at +1 versus +6 hours of starvation; confusingly, these mechanisms are partially distinct from those that contribute to differences in negative thermotaxis behaviors in well-fed and +3 hours of starvation worms (Takeishi et al, 2020). A limitation of this manuscript is that these differences are not particularly acknowledged or addressed, other than the hypothesis that independent mechanisms underlie negative thermotaxis versus thermonociceptive stimuli. However, this suggestion is not experimentally verified.

We thank this reviewer for pointing to the interest of our work. The difference between previous work focusing on negative thermotaxis in the range of innocuous temperatures and our work focusing on thermo-nociceptive response is important and indeed deserves further clarification and a deeper discussion in the manuscript.

Two major empirical evidence for a distinction between negative thermotaxis (as assessed in previous studies) and thermonociceptive plasticity (as assessed in our paradigm) were already included in the initial article version. First, we reported a decrease in average response in AWC neurons due to a shift in the distribution of response polarities from mostly up-response to a mix of ‘up-response’ and ‘downresponse’ after starvation, while previous results showed an increase in response probability of AWCs after starvation (Takeishi et al, 2020). Second, contrary to starvation-evoked thermotaxis adaptation, ASI neurons are required to orchestrate starvation-evoked plasticity in thermonociception. These observations already indicate differences at the circuit and cellular level. For the revision, we conducted further experiments to address the molecular level. We tested *ins-1* mutants (see also specific point 3 by reviewer 2, below) and deepened this aspect in the discussion section of the revised manuscript. Previous study found that INS-1 signaling from the intestine is a major mediator of negative thermotaxis plasticity. In contrast, our new data show that INS-1 peptide does not seem critical in regulating starvation-dependent thermonociceptive plasticity (see new Figure 6-supplement 1). Taken together these three lines of empirical evidence support the notion that negative thermotaxis and thermonociceptive starvation-evoked plasticity involves at least partially distinct mechanisms and it seems therefore inappropriate to qualify this notion as purely hypothetical.

Modification in the revised manuscript include extended introduction about the known thermotaxis regulation mechanisms (Introduction section), new Figure 6-supplement 1 about *ins-1* and accompanying text in the result section as well as extended discussion about these differences (Discussion section)

Multiple additional aspects of this study make the results difficult to synthesize with existing knowledge, including

(1) Differences in - and insufficient discussion of - the magnitude and kinetics of thermal stimuli;

We have included a better description of the stimuli characteristics in the revised methods section. The discussion section was deepened to better emphasize that different types of thermal stimuli have been used in different studies.

(2) This study's use of "heating power" rather than temperature values when presenting behavioral results;

Thanks for noting this point, which was indeed an unnecessary complication in the result display of the initial manuscript. We have changed ‘power values’ to corresponding ‘temperature increase’ in the revised figures.

(3) The use of +1 hours starvation as a baseline instead of well-fed worms. Indeed, this last point reflects a noticeable experimental result that differs from previous studies, namely that at room temperature, the basal movements of well-fed and starved worms are not different. Such a surprising result warrants further quantification of worm mobility in general and could have prompted a set of experiments directly testing previously published thermal conditions to demonstrate that the new effects reported arise specifically from the use of thermonociceptive stimuli, as hypothesized.

The consideration of on-food and off-food behavioral state is an important point indeed. We found that, at room temp, worms shift from dwelling on food (a state with high spontaneous reversal rate) to global search off-food (a state with low spontaneous reversal, after 1hr starvation) (see Figure 1B). Therefore, unlike the reviewer’s statement, the reported data highlighted key differences in the basal locomotion of worms in fed and 1hr starved conditions. Furthermore, these behavioral states have been characterized very deeply using high-content worm behavioural tracking in our recent publication: Thapliyal et al. 2023 (PMID: 37236963). Our choice of using 1hr as a baseline is primarily driven by the fact that an elevated baseline of spontaneous reversals on food decreased the dynamic range to monitor changes in heat-evoked reversals. 1-hour early food deprivation reduced spontaneous reversals and led to a mild attenuation of heat-evoked responses at low stimulus intensities, while responses to stronger stimuli remained comparable to those of fed animals. Additionally, we observed a clear progressive decrease in heat-evoked reversals with increased duration of food-deprivation, which we further used to dissect the mechanism underlying this plasticity. The choice of the 1hr food deprivation timepoint as a reference is further justified below.

Finally, a previous report (Yeon et al, 2021) demonstrated differences in the impact of chronic versus acute neural silencing on starvation-dependent plasticity in the context of negative thermotaxis. We therefore wonder whether similar developmental compensation impacts the neural circuits that contribute to starvation-dependent plasticity in the thermonociceptive responses.

Indeed, this is an interesting question. Our conclusions are so far based on ablation (with chronic effects). In order to gain insight on this question, future studies could address the impact of chronic vs acute silencing approach in starvation-dependent thermonociceptive responses. We have added an opening on this question in the discussion, as follows:

“Another open question is whether ASI action takes place during development (prior to starvation), or more acutely with active signaling after starvation.”

A weakness of this manuscript is that the introduction is insufficiently scholarly in terms of citations and the description of current knowledge surrounding the impact of internal state on sensory behavior, particularly given previous work on the impact of feeding state on thermosensory behavioral plasticity (Takeshi et al 2020, Yeon et al 2021) and chemosensory valence (Banerjee et al 2023, Rengarajan et al 2019, etc).

To address this weakness, we have revised the introduction section of the manuscript and cited previous relevant research on the impact of internal states on animal behavior, including the papers suggested by the reviewer. We note that 2 out of 4 suggested citations were already present in the initial manuscript (though in the discussion section).

Similarly, the authors' commanding knowledge of the distinction between thermotaxis navigation (especially negative thermotaxis) and thermonociceptive behaviors could be communicated in more depth and clarity to the readers, in order to contextualize this study's new findings within the previous literature.

As mentioned above, we have deepened this aspect in the discussion section of the manuscript (with a dedicated paragraph). It is quite clear that starvationinduced plasticity in negative thermotaxis and thermonociceptive behaviors engage distinct mechanisms (at least in part). These differences include distinct alterations in AWC calcium activity, role of ASI neurons and INS-1 neuropeptide.

Nevertheless, this study represents a solid addition to the growing evidence that *C. elegans* sensory behaviors are strongly impacted by internal states, and that neuropeptidergic signaling plays a key role in mediating behavioral plasticity. To that end, the authors have provided solid evidence of their claims.

We thank this reviewer for the efforts in evaluating our manuscript, for the positive assessment of our work, and for highlighting some weaknesses which, we believe, have been addressed through the revision.

**Reviewer #2 (Public review):**

In this work, Thapliyal and Glauser tried to provide a mechanistic understanding by which animals modulate their neural circuit responses to control nociceptive behavior on the basis of the dynamic internal feeding state. It is an important study that adds to the growing body of evidence coming from multiple model systems. They have used elegant genetics, behavioral, and Ca-imaging experiments to demonstrate how the auxiliary thermosensory neuron pair, AWC, and one of the internal state-sensing interneuron pairs, ASI, respond to dynamic internal starvation state to modulate behavioral response to noxious heat. Interestingly, these neuron pairs use distinct molecular mechanisms along with some other unidentified neurons to suppress heat-induced reversal response under short-term and prolonged starvation. The experiments are well performed, supporting most of the claims and providing an important framework for future studies.

I have some queries that, if answered, will certainly enhance the study.

(1) The results suggest that ASI is one of the primary drivers for the starvation-evoked behavioral plasticity, which regulates AWC activity under prolonged starvation. It raises many important questions, including: (a) how starvation modulates ASI response to heat?, and (b) under prolonged starvation, whether ASI also promotes other, non-AWC, glutamatergic inhibitory neurons to suppress heat-induced reversal, and how?

We agree with this reviewer that the mechanisms by which ASI detects and mediates starvation-evoked changes in our model is a very interesting (unsolved) question. However, addressing these questions empirically represents a substantial body of work that would go beyond the scope of the present report. E.g., is temperature-dependent activity in ASI even relevant? At present, we envision that ASI could either work acutely (during heat stimuli) or be modulated over much longer time frames (hours of starvation) as an internal state sensor. Therefore, there will be quite some exploration needed before we figure out the ASI-level regulation more fully (including the critical temporal aspect regarding cell activity, as well as quantitative and qualitative transmission aspects). It will be very interesting in future work to address these questions.

(2) How does ASI regulate AWC activity? In the proposed model (Figure 8) authors suggested an independent, unknown signal, other than INS-32 and NLP-18, from ASI to regulate AWC activity. However, from the results, the existence of another signal is not very clear.

Thanks for raising this point, which reveals a weakness in our graphical representation (in Fig. 8) that was not properly conveying our point. Our current work shows INS-32 and NLP-18 to be important in modulating heat-evoked reversals upon starvation. However, at the moment, we don't know if INS-32, NLP-18, both, and/or other neuropeptides from ASI modulate AWC activity patterns. The calcium imaging experiments in single, double and potentially triple mutants would answer these questions but are not within our current reach, given the time needed to carry out these experiments. However, we acknowledge this point and have changed the figure and its legend to state that the arrow connecting ASI to AWC activity pattern could potentially reflect the action of these neuropeptides.

(3) Previously, Takeishi et. al. showed that ins-1 dynamically modulates AWC-AIAmediated thermotaxis behavior based on the feeding state of the animal. It raises questions whether ins-1 also contributes to noxious heat-induced reversal behavior.

We thank the reviewer for this question. We have now quantified the phenotype of *ins-1* mutant in our paradigm. Our data shows that INS-1 neuropeptide is not critical in mediating starvation-evoked thermonociceptive plasticity, unlike plasticity in thermotaxis behavior (See Figure 6- Supplement 1). Together with the differential activity patterns in AWC and the differential need for ASI neurons, these new data further consolidate the notion that starvation-evoked thermotaxis adaptation and noxious-heat avoidance engage separable molecular, cellular and circuit-level modulatory mechanisms. A specific discussion paragraph was added too.

(4) Experiments with AWC fate conversion mutants (nsy-1 and nsy-7) were very good ideas; however, the results obtained were confusing. flp-6 mutant data suggest AWCoff would be essential for heat-induced reversal, especially at the low intensity stimulus level. However, the nsy-1 mutant-forming two AWCon neurons showed complete rescue at the low heat level, which is quite opposite. Similarly, although less prominent, eat-4 rescue experiments suggested both nsy-1 and nsy-7 should behave normally at high heat conditions, which was not the result observed.

We appreciate this comment and the legit attempt to infer what we should expect from a worm with two AWCon or two AWCoff, respectively. From previous studies so far, it's not quite clear if cellular properties of newly formed AWCs in *nsy-1* and *nsy-7* mutants, including response to sensory cues, formed synapses and their partners, expression of neuromodulator and gap junctions, synaptic output are similar or different. We think further studies are required to first establish if FLP-6 and glutamate signaling (expression, release and action) from altered AWCs in *nsy-1* and *nsy-7* mutants are the same or different. Therefore, direct comparison between cell fate conversion mutants with *flp-6* and glutamate would rely on too many assumptions at this stage. Considering this comment, the limited additional value of the data with *nsy1* and *nsy-7* mutants (in the absence of additional analyses) and the confusion it could trigger, we have decided to remove these non-essential data of the manuscript.

**Reviewer #3 (Public review):**

Summary:

Thapliyal and Glauser show that hunger alters how *C. elegans* responds to noxious thermal stimuli. Using targeted neural ablation, mutant analysis, and live-cell functional imaging, the authors demonstrate that hunger changes the properties of AWC sensory neurons, which sense noxious heat. The authors further show that the effects of hunger on nociception require ASI neurons, which are known to respond to hunger and mediate the effects of food deprivation on behavior. Finally, the study uses mutant analysis to implicate glutamate and specific neuropeptides in thermal nociception and in the modulation of nociceptors by hungerresponsive neurons.

Strengths:

The study clearly shows a strong effect of hunger on nociception and documents a striking effect of hunger on the intrinsic properties of AWC sensory neurons, which respond to noxious heat. The study also clearly and compellingly demonstrates that ablation of hunger-responsive ASI neurons blocks the effects of hunger on nociceptive AWCs. These data, which constitute the kernel of the manuscript, are striking and exciting.

Weaknesses:

The study has some weaknesses that the authors should address.

(1) Ablation of AWC neurons alters the basal sensitivity to noxious heat stimuli. This should be clearly noted in the description of the result and warrants some discussion.

We thank this reviewer for raising this legitimate point. We have clarified this aspect in the results section of the revised manuscript, reading as follows:

“Removal of AWC nearly abolished heat-evoked reversal behavior across all stimulus intensities and timepoints (Figure 2B and E). While one should keep in mind that potential indirect developmental effects might take place in neuro-ablation lines, this observation suggests that AWC plays an essential role in mediating the thermonociceptive response under both early food deprivation and prolonged starvation. Notably, in AWC-ablated animals, the residual response level was unaffected by starvation, suggesting that AWC might also be required for the expression of starvation-dependent plasticity.”

The contrast with known function of the best-characterized sensory neurons mediating thermal nociception (AFD and FLP) is discussed as follows:

“...Therefore, noxious heat-evoked activity in AWC varies widely according to context, which is in line with previous literature [18, 20, 41]. Interestingly, the role of AWC is distinct from that of AFD and FLP neurons, which are canonically linked to thermosensation and nociception [5, 14, 42, 43], but contribute only modestly to heat-evoked behavior in our assay conditions with between 1 and 6 hrs of food deprivation.”

(2) Throughout the study, it seems that data are replotted in multiple figure panels. The authors should clearly indicate in the figure legends when this occurs. Also, the authors should ensure that statistical tests requiring multiple comparisons are correctly implemented and reflect the number of times experimental data are compared to a single set of control data.

Thanks for raising this important point. We have clarified this aspect in the revised figure legends of the manuscript, and in the method section. In some instances, we reconducted some analyses to be perfectly rigorous in multiple comparison accounting. This did not lead to significantly different conclusions. The one exception was that the small effect of *eat-4* mutation on spontaneous reversal went below significance threshold. We therefore removed this aspect of the result reporting and of the corresponding interpretation scheme, which became slightly simpler (Figure 3). Globally, this makes the story more focused.

(3) How ASIs modulate AWCs remains unclear. The authors find that loss of INS-6, an insulin-like peptide provided by ASIs, partially recapitulates the effect of ASI ablation. This observation is not further developed, and instead, the authors characterize other secreted factors that seem to mediate sensitization of animals to noxious heat stimuli. While it is interesting that there are multiple opposing inputs into the nociceptor circuit, the essential connection between ASIs and AWCs that underlies the foundational observations in Figures 1 and 2 is not sufficiently characterized.

Whereas we agree that how ASI modulates AWCs is only partially solved by our study, we should emphasize that our work identified two ASI-expressed neuropeptides that function to decrease reversal response after starvation: INS-32 and NLP-18. We initially set a lower priority on INS-6 because the reversal response level in starved mutants appeared lower than that in *nlp-18* and *ins-32*. It is important to note that *ins-32* and *nlp-18* are not ‘generally potentiated’ mutants, but display reversal upregulation selectively following starvation, which placed them as strong candidates to selectively mediate ASI regulation. This said, it is also true that these two mutants (and *ins-6* too) display reduced responsiveness at the early food deprivation time point. Therefore, none of the neuropeptide mutants was strictly identical to ASI ablated line, suggesting that the peptides might also work via non-ASI cells at the early food deprivation timepoint.

Following this reviewer’s comment, we have attempted to complement our story with the idea of using a similar approach and rescue *ins-6* with its endogenous promoter or ASI-specific promoter. Unfortunately, we failed to obtain rescue effects, and therefore these data (with a negative result) remain inconclusive (as we cannot guarantee that the rescue constructs were functional). We decided to keep these data aside in the revised manuscript. Globally, our point made graphically in Figure 7F remains valid. We have complemented the figure legend to mention that INS-6 could also potentially work from ASI, but it is not depicted as no ASI-specific data are available. In summary, our data suggests that the connection between ASI and AWC(s) might be established by the integrated action of multiple peptides and their receptors. Further calcium imaging experiments in single, double and potentially triple mutant(s) of peptides and receptors would be required go deeper in this question, which could be performed in future work.

“...Additional neuropeptides (such as INS-6) may also be involved, but in the absence of direct evidence for their origin from ASI, they were not included in this scheme.”

(4) The assertion that 'starvation reshapes AWC responses from deterministic to stochastic' is not clearly supported by the data. AWC neurons seem capable of showing different responses to thermal stimuli, and the probabilities associated with these responses change after fasting. The different kinds of responses are seen under basal and fasted conditions.

We thank this reviewer for the comment. There is an activity response shift that is quite solidly described, including with new quantitative analyses of distributions (histograms in new Fig. 4CD and new Fig. 5C-D, accompanied by Kruskal-Wallis tests). Yet, we totally agree that the wording choice was inappropriate. We have furthermore changed our terminology to avoid using the terms “stochastic” or “deterministic” that were indeed a cause of confusion. We now use the terms “stimulus-locked responses” and describe the shift as “shift from mostly excitatory responses to a mix of both excitatory and inhibitory responses”. We have also included detailed methodology for characterization of traces and statistical analysis in the revised method section of the manuscript, together with the new analyses on peak polarity distribution.

**Recommendations for the authors:**

**Reviewing Editor Comments:**

The reviewers agree that the study is clearly presented and makes good use of behavioral, genetic, and imaging approaches to link starvation state with changes in AWC and ASI function. To strengthen the manuscript and ensure clarity for readers, we ask you to address the following points in revision:

(1) Positioning and citations.

Clarify how your findings relate to Takeishi 2020, where the opposite trend in AWC activity was reported, and make a clear distinction between thermonociception and thermotaxis. The introduction should also include additional citations in two specific areas: prior work on AWC and noxious thermal stimuli, and studies demonstrating starvation-dependent behavioral changes via altered neuropeptide release (e.g., Banerjee 2023; Rengarajan 2019).

We have clarified this aspect with extension of the work cited in the introduction and extensive rewriting of the discussion sections.

Our data shows that mechanisms underlying starvation dependent changes in thermonociception and thermotaxis show differences at the molecular, cellular and circuit levels. First, we see a decrease in average response in AWC neurons due to shift from mostly excitatory to a mix of excitatory and inhibitory responses in response to noxious heat after starvation, while previous study found an increase in response probability of AWCs after starvation (Takeishi et al, 2020). Second, contrary to thermotaxis behavior ASI neurons are required to orchestrate starvation evoked plasticity in thermonociception. And, finally, previous study found that INS-1 signaling from the intestine regulates thermotaxis behavioral plasticity while INS-1 peptide does not seem critical in regulating starvation-dependent thermonociceptive plasticity (new data in Figure 6 supplement 1).

We have revised the introduction section of the manuscript and cited previous relevant research on AWC and noxious thermal stimuli and studies demonstrating starvationdependent behavioral changes via altered neuropeptide release including the papers suggested by reviewers. The extended discussion section reads as follows:

“Starvation regulates thermonociceptive and negative thermotaxis plasticity via at least partly different mechanisms

Previous studies showed that AWC plays an important role in starvationdependent plasticity in the negative thermotaxis behavior in an innocuous thermal range between 15 and 25°C [26, 33]. Negative thermotaxis involves the detection of thermal changes created by animal movement in spatial thermogradient (0.5°C/cm), the magnitude of the expected thermal changes approximating 0.01°C/s [26]. The starvation impact on negative thermotaxis was shown to (i) involve an up-regulation of AWC cell activity, (ii) rely on INS1 neuropeptide produced in the intestine and (iii) to occur independently of ASI neurons. In contrast, our study used thermo-nociceptive stimuli, with faster raising thermal slopes (~0.5-2°C/s, hence 50-200 times faster than those occurring for thermotaxis) and covering noxious temperatures (up to 28°C). Our results indicate that the regulation of thermo-nociceptive response by starvation (i) is linked to a shift in the distribution of AWC activity response polarities from mostly excitatory to a mix of excitatory and inhibitory response, (ii) relies on ASI and specific neuropeptide produced in ASI, and (iii) works independently of INS-1 neuropeptide. Therefore, our study complements our understanding of the modulation of temperature-dependent behavior in *C. elegans* with previously undocumented mechanisms at the circuit, cellular and molecular levels.”

(2) ASI → AWC mechanism.

Because ASI is central to your conclusions, please expand on how ASI is thought to act on AWC and/or other neurons. If an additional ASI signal is proposed beyond INS-32/NLP-18, mark this as speculative unless further rationale can be provided, and adjust the model figure accordingly.

We have revised Figure 8 and its legend to clarify what is still hypothetical in the way ASI could affect AWC activity patterns and reversals. Note that the figure was also modified to integrate the conclusions made from epistasis analysis of *eat-4* and *flp-6*.

(3) AWC response description.

The data support a shift in response distributions rather than a categorical switch from "deterministic to stochastic." Please adjust the language accordingly and provide a clear description of how traces were classified as "up, variable, or down," ideally with a quantification of the distributional shift.

We agree that the term “stochastic” can convey different things, and because it was used for something different for AWC in the past, we should have avoided it. We have revised the nomenclature. What we observe can indeed be better described as a shift in the response polarity distribution. The article was revised accordingly. We also included the quantitative analysis and histogram representation, suggested in one of the specific comments, and added detailed methodology on the categorization of traces.

When ASI is intact, we see a shift from mostly excitatory responses to an ~equal mix of excitatory and inhibitory responses (new Figure 4C-D). This effect is lost when ASI is ablated (new Figure 5C-D).

(4) Presentation and statistics.

In figure legends, indicate where datasets are replotted across panels and confirm that multiple-comparison corrections take account of repeated comparisons to the same controls.

We have included these details in the revised figure legends, and a statement in the method section.

(5) Methods clarity.

Provide justification for using 1-h off-food as the baseline, with quantification of baseline mobility/reversal rates. Expand the calcium-imaging methods to describe the processing pipeline (ΔR calculation, baseline period, drift correction), and add a brief rationale if the approach deviates from common normalization procedures. Clarify how cell ablations were performed and verified for specificity, and how cell-specific rescues were confirmed. Please also acknowledge the potential for developmental compensation with chronic ablation.

The justification of using 1hr off-food as baseline was made more prominent in the revised manuscript.

Revised result section:

“Starvation downregulates thermonociceptive responses in *C. elegans*

To assess how the feeding state modulates thermonociceptive behavior in *C. elegans*, we compared responses across different durations of food deprivation (Figure 1A). Synchronized first-day adult animals were stimulated with a series of 4-s infrared pulses of increasing heating power (100, 200, 300, 400 W), causing temperature increase of +2°C, +4°C +6°C and 8°C at the surface of the plate (Figure 1A). Fed animals on food produced robust heat-evoked reversal response to heat, but they also displayed a very elevated baseline of spontaneous reversals (~38%). A 1-hour off-food condition reduced spontaneous reversals (from ~38% to ~10%) and led to an attenuation of heat-evoked responses at low stimulus intensities, while responses to stronger stimuli remained comparable to those of fed animals. More prolonged food deprivation led to a striking progressive reduction in thermonociceptive responses at every heating level, with responses after 6 hours of starvation approaching baseline spontaneous reversal rates (Figure 1B and C). This suggests a robust inhibition of nociceptive behavior caused by prolonged starvation. To determine whether this attenuation was due to the absence of nutrients or chemosensory cues, we conducted similar starvation experiments in the presence of food odor, with OP50 bacteria present on the petri dish lid (Figure 1D). The reduction in thermonociceptive response persisted, indicating that the effect is driven by the internal starvation state rather than external olfactory input.

Although fed animals showed high sensitivity to noxious heat, they also displayed an elevated baseline of spontaneous reversals, which limited their utility as a control group by strongly reducing the dynamic range of heat-evoked reversal quantification and by complicating the quantitative comparison with food-deprivation conditions with much-reduced reversal baseline (Figure 1B). In addition, technical limitations in our calcium imaging setup would have prevented the intended follow-up analyses in fed animals. Based on these observations and technical considerations, we focused subsequent analyses, aiming at dissecting the circuit and molecular underpinnings of starvation-dependent plasticity, to the comparison of two off-food conditions with similar spontaneous reversal baseline: the early food deprivation condition (1-hour off-food, with high responsiveness to noxious heat) and the prolonged starvation (6-hour off-food with almost abolished noxious heat responsiveness).”

In addition, the method section was modified as follows:

- Calcium imaging details were added regarding ΔR calculation, baseline period, drift correction.

- We now explicitly refer to the original respective articles describing the neuroablation lines.

- We clarify that cell-specific transgene expression for rescue was confirmed using SL2::mCherry co-marker

In the result section, we now explicitly address potential developmental compensation in genetic ablation backgrounds in the result section as follows: “…one should keep in mind that potential indirect developmental effects might take place in neuro-ablation lines”.

**Reviewer #1 (Recommendations for the authors):**

(1) The data availability statement is missing from the reviewed manuscript and should be included.

Thanks, we have included the data availability statement in the revised manuscript.

(2) We request additional information on how n's were determined for individual experiments, as well as the inclusion of post-hoc power measurements for all quantification.

n were determined in agreement with previous studies using similar measures. No a priori power analyses were performed. A posteriori power analyses are not informative beyond the reported effect sizes and p-values (now reported in File S2). We clarified this in the statistical subsection of the method section.

(3) In many cases, the specific statistical tests used are not clear or justified; more details should be provided, including the non-post-hoc test used. Are all tests one-way ANOVAs? For comparisons across genotype and starvation duration, two-way ANOVAs would likely be more appropriate. Also, the authors switch between Bonferroni post-hoc tests and Holm-Bonferroni post-hoc tests. What determined the use of one versus another?

We have now clarified the statistical analyses used and provided full details in File S2. We have now more systematically applied two-way ANOVAs across all relevant analyses (with detailed parameters reported in File S2). When particularly relevant (e.g epistasis analysis between *eat-4* and *flp-6* mutations) the results of the two-way ANOVAs, is also explicitly stated in the result section.

We also note that all multiple-comparison corrections were performed using the Bonferroni method. Previous mentions of Holm-Bonferroni correction were inaccuracies, and we apologize for this confusion; these mentions have now been corrected throughout the manuscript.

(4) The use of heating power instead of the temperature experienced by the worms is an unwelcome abstraction. We strongly recommend revisiting that choice.

We do agree. We have revised the figures to label the axis with temperature increase.

(5) For calcium imaging, how are the traces categorized into "calcium up", "calcium down", or "no change"? Were those determined blindly - i.e., by individuals unaware of the experimental condition? Did the response direction need to be consistent across different temperatures? Did the change from baseline need to hit a specific threshold, consistent with previous studies in the field (i.e., +/- 3xSD for a minimum amount of time)? We encourage the authors to include these details in their methods section.

We have complemented the method section to clarify the criteria for the qualitative classification of traces. More importantly, new quantitative peak polarities comparisons were added (see specific points below and above, about histograms).

(6) For the experiments showing that exposure to food odor does not prevent response reduction, we suggest that feeding worms heat-killed bacteria would be a helpful control for the importance of bacterial nutritional status. In addition, showing that the impact of starvation was reversible with re-feeding would have been a useful experiment in line with standard experimental design in the starvation field.

Thanks, indeed with our current work we cannot pinpoint the role of additional sensory cues (except food odor) to be mediating starvation-evoked plasticity. Together with refeeding, these are all extremely interesting questions that we aim to answer and potentially link with ASI and AWC activity in our future work.

(7) For the various AWC rescue experiments, we found it curious that there wasn't an AWCon+off rescue, only each neuron individually.

Previous studies have identified similar or opposite responses of both AWCs for distinct sensory cues. Though our calcium imaging experiments point to both AWC on and off having similar response patterns to heat, we cannot rule out the possibility that their output (ability of control reversals) is distinct possibly due to recruited neuromodulators. Therefore, in the present work, we examined where these cell types act via the same or distinct combinations of neuromodulators to control reversals.

**Reviewer #2 (Recommendations for the authors):**

Experiments suggested:

(1) The authors should look into the Ca-dynamics in ASI.

How does the spontaneous and heat-evoked activity of ASI differ in fed, early food-deprivation and prolong starvation and its link to releases of neuromodulators, modified AWC activity to alter output of thermal nociception are very interesting questions. However, these questions are extremely exploratory (see argumentation above in response to the public review) and addressing them goes beyond the scope of our current manuscript.

(2) The authors should check AWC activity in ins-32 and nlp-18 mutant animals.

In this study, we focused on the roles of INS-32 and NLP-18 released from ASI in modulating heat-evoked reversals, as these mutants exhibit relatively strong behavioral phenotypes. However, these effects remain less pronounced than those observed following ASI ablation. In addition, we cannot exclude the contribution of additional signaling molecules, including INS-6 and other neuropeptides.

A comprehensive analysis of AWC activity in this context would require calcium imaging across multiple genetic backgrounds, including single, double, and potentially higher order peptide and receptor mutants, combined with cell-specific rescue experiments. While we appreciate the suggestion, such an approach would represent a substantial extension of the present work and will be important to pursue in future studies to further elucidate the underlying mechanisms.

(3) Short-term food deprivation completely eliminated heat heat-induced reversal response to 100W stimulus, while the response to 400W stimulus remained unaffected. This suggests fed, short-term starvation, and prolonged starvation are three distinct states, and authors should also test the response of AWC and ASI ablated animals in the fed conditions.

We agree that analyzing thermal nociception in fed states, in addition to short-term and prolonged food deprivation states is an important and interesting question, as these 3 conditions likely represent 3 distinct internal states that may recruit different neural pathways.

Several reasons led us to set the fed condition aside for this study, and we realize we insufficiently explain them in the initial manuscript. There are 2 main reasons.

(1) It is of paramount importance to consider the ‘baseline’ reversal rate (spontaneous reversals not triggered by heat, but visible in our dataset as the first point in the ‘dose-response’ curve). In Fed animals spontaneous reversal rate is very high (~38%) compared to the 1hr and 6hr food-deprivation conditions (>10%). This has two consequences: first a decreased dynamic range for quantify heat-evoked reversal, and, second, the difficulty in judging quantitative differences in heat-evoked reversals with such major differences in baseline reversals.

(2) Experimentally, assessing calcium responses in truly fed animals presents technical challenges. With our current setup, animals must be removed from food for at least ~5 minutes prior to recording (followed by ~5 minutes of imaging), which effectively corresponds to a “freshly starved” condition rather than a fully fed state. While previous studies have used serotonin to mimic aspects of the fed state, such manipulations can be difficult to interpret in this context.

A systematic comparison including fully fed animals, as well as AWC- and ASI-ablated conditions across these states, would be a valuable direction for future work, in particular once the methodological barriers associated with point 2, have been overcome.

We have clarified these choices in the result section as follows:

“Starvation downregulates thermonociceptive responses in *C. elegans*

To assess how the feeding state modulates thermonociceptive behavior in *C. elegans*, we compared responses across different durations of food deprivation (Figure 1A). Synchronized first-day adult animals were stimulated with a series of 4-s infrared pulses of increasing heating power (100, 200, 300, 400 W), causing temperature increase of +2°C, +4°C +6°C and 8°C at the surface of the plate (Figure 1A). Fed animals on food produced robust heat-evoked reversal response to heat, but they also displayed a very elevated baseline of spontaneous reversals (~38%). A 1-hour off-food condition reduced spontaneous reversals (from ~38% to ~10%) and led to an attenuation of heat-evoked responses at low stimulus intensities, while responses to stronger stimuli remained comparable to those of fed animals. More prolonged food deprivation led to a striking progressive reduction in thermonociceptive responses at every heating level, with responses after 6 hours of starvation approaching baseline spontaneous reversal rates (Figure 1B and C). This suggests a robust inhibition of nociceptive behavior caused by prolonged starvation. To determine whether this attenuation was due to the absence of nutrients or chemosensory cues, we conducted similar starvation experiments in the presence of food odor, with OP50 bacteria present on the petri dish lid (Figure 1D). The reduction in thermonociceptive response persisted, indicating that the effect is driven by the internal starvation state rather than external olfactory input.

Although fed animals showed high sensitivity to noxious heat, they also displayed an elevated baseline of spontaneous reversals, which limited their utility as a control group by strongly reducing the dynamic range of heat-evoked reversal quantification and by complicating the quantitative comparison with food-deprivation conditions with muchreduced reversal baseline (Figure 1B). In addition, technical limitations in our calcium imaging setup would have prevented the intended follow-up analyses in fed animals. Based on these observations and technical considerations, we focused subsequent analyses, aiming at dissecting the circuit and molecular underpinnings of starvationdependent plasticity, to the comparison of two off-food conditions with similar spontaneous reversal baseline: the early food deprivation condition (1-hour off-food, with high responsiveness to noxious heat) and the prolonged starvation (6-hour off-food with almost abolished noxious heat responsiveness).”

(4) The authors should test the effect of ins-1 in noxious heat-mediated dynamic reversal behavior.

We thank the reviewer for this valuable suggestion. We have now tested the phenotype of *ins-1* mutants in our paradigm. Our data shows that INS-1 neuropeptide is not critical in mediating starvation-evoked thermonociceptive plasticity unlike plasticity in thermotaxis behavior (New Figure 6 Sup1). This molecular aspect adds to our initially presented evidence at the cell activity and circuit levels, that noxiousevoked reversal and thermotaxis behaviors are regulated in a clearly separable manner.

(5) Whether Glutamate and flp-6 work in parallel or in the same pathway to regulate reversals?

We thank the reviewer for this question. We tested *eat-4*; *flp-6* double mutants and found:

(1) After short-term food deprivation (1hr), *flp-6* and *eat-4* separately contribute to heat-evoked reversal at high & low heat and they act in parallel pathways to explain ~90% of animal responsiveness (new version of Fig 3)

Corresponding new text:

“Next, we focused on *eat-4* and *flp-6* mutants, showing the strongest phenotype. We addressed whether glutamate and FLP-6 signaling act dependently of each other in controlling heat-evoked reversal, by testing *eat-4*; *flp-6* double mutants. The residual response seen in each single mutant (Figure 3 A and B) was almost entirely abolished in the double mutant (Figure 3C). A two-way ANOVA for the highest heat stimuli with *eat-4* and *flp-6* genotypes as factors (two levels each: mutant or wild type) showed significant main effects of *eat-4* (*F_(1,67)_* = 48.70, *p*<.001, *η^2^p*=0.421) and *flp-6* (*F_(1,67)_* = 58.50, *p*<.001, *ηp*=0.466), respectively, but no interaction effects (*F_(1,67)_* = 0.093, *p*=.761, *η^2^p*=0.001). The significant cumulative effect of the two mutations indicates that the two signaling pathways act mostly independently of each other to mediate heat-evoked reversals.”

(2) After prolong starvation (6hr), *flp-6* mutation has a dominant impact on plasticity and *eat-4* mutation cannot cause loss of plasticity, pointing to a hierarchy in this context (new version of Fig. 6, including revised hierarchy in the model in panel G, and also revised model in Fig.8).

Corresponding revised text:

“Second, we tested whether starvation-dependent plasticity was preserved in *eat-4* and *flp6* mutant backgrounds, which we had suggested to represent the main AWC transmitters controlling heat-evoked reversals under the early food deprivation condition (Figure 3). Even if the heat-evoked response upon early food-deprivation was reduced relative to wild type in *flp-6* mutants, a significant further decline was seen after prolonged starvation (Figure 6B). These results indicate that starvation-dependent plasticity can operate independently of FLP-6. In contrast, *eat-4* mutants displayed markedly elevated heat-evoked responses after prolonged starvation, even exceeding the response level seen in the early food deprivation condition for low heat stimuli (Figure 6C). This potentiated response in *eat-4* mutants was entirely dependent of an intact FLP-6 signaling, since reversal responses in *eat-4*; *flp-6* mutants were entirely abolished, like in *flp-6* single mutant (Figure 6C-E, a two-way ANOVA indicating a significant interaction effect between the two mutations: *F_(1,70)_* = 0.093, *p*<.001, *η^2^p*=0.247). Moreover, the potentiated response in *eat-4* single mutant could not be rescued by expressing *eat-4* rescue transgene selectively in either AWC^OFF^ or AWC^ON^ neurons (Figure 6F). Interestingly, AWC^OFF^-specific rescue produced a further potentiation of heat-evoked reversal response to high heat stimuli (Figure 6F, 6 and 8°C thermal increases), aggravating the phenotype of *eat-4* mutants. These results are consistent with a model in which glutamatergic signaling regulates heat-evoked reversals in starved animals via two bidirectional drives (Figure 6G). On the one hand, glutamatergic signaling—originating from AWC^OFF^—up-regulates reversals in response to high heat stimuli, thus contributing to prevent starvation-induced thermonociceptive plasticity. On the other hand, glutamatergic signaling—originating from neurons other than AWC— down-regulates reversals over a broad range of heat intensities, thus promoting starvation-induced thermonociceptive plasticity. The latter glutamatergic signaling inhibitory effect seems to be more dominant and to depend on intact FLP-6 signaling.”

(6) The authors should perform flp-6 and eat-4 mutant/rescue experiments in the nsy-1 and nsy-7 background to clarify the results.

Our results indicate that glutamate release via EAT-4 from both AWC^ON^ and AWC^OFF^, as well as FLP-6 from AWC^OFF^, contributes to heat-evoked reversals regulation. The experiments suggested by the reviewer would, in principle, provide further insight into the interaction between AWC subtype identity and the respective roles of glutamatergic and peptidergic signaling.

However, as discussed in more details above in the public review, the extent and nature of AWC^ON/OFF^ remodeling in *nsy-1* and *nsy-7* mutant backgrounds remain incompletely understood. This introduces significant uncertainty in interpreting results obtained from combining these mutations with *eat-4* and *flp-6* manipulations. As a result, such experiments would be difficult to interpret in a definitive manner at this stage.

We therefore consider this an important direction for future work, once the roles of *nsy1* and *nsy-7* in AWC subtype specification and function are more clearly established. As our preliminary results with *nsy-1* and *nsy-7* mutants added more confusion than clarity, we have chosen to set them aside (former Fig. 3-figure supplement 2 has been removed).

Minor comments:

(1) What is food odor? The experiment should be clearly mentioned.

Thanks for spotting this unintended omission. Food odor experiments were performed by adding OP50 bacteria on the inward side of the petri dish lid instead of the NGM surface. We have added this description in the method section of the revised manuscript.

(2) Panel 3C is coming before 3B. This should be rearranged.

Thanks for pointing this out. Panel arrangement was entirely reorganized in revise Fig. 3, with the addition of *eat-4 x flp-6* genetic interaction analysis.

(3) In Figure 6, if the panels are arranged horizontally, it would be easier to follow.

Thanks for pointing this out. Panel arrangement was entirely reorganized in revise Fig. 6, with the addition of *eat-4 flp-6* genetic interaction analysis.

**Reviewer #3 (Recommendations for the authors):**

(1) The authors should consider moving measurements of AFD-ablated animals into the main Figure 1. AFD is a well-known thermosensor, and it is worth showing that responses to noxious thermal stimuli persist in animals lacking AFD.

Thank you for this suggestion. We have moved measurements of AFDablated animals to the main figure (revised Fig. 2).

(2) AFD ablation does affect responses to noxious heat. The authors could consider ablating/silencing AFDs and AWCs simultaneously to determine whether these two neurontypes account for the behavior.

We agree that investigating the combinatorial contributions of thermosensory neurons, including AFD and AWC, to thermal nociception is an important and interesting question. In principle, simultaneous ablation or silencing of these neuron types could indeed reveal unexpected interactions.

In our experimental paradigm, however, we observe only a minimal contribution of AFD neurons to heat-evoked reversals, whereas ablation of AWC nearly abolishes the response. Based on these observations, we chose to focus the present study on AWC, which appears to play a more prominent role in this behavior.

A more detailed dissection of the potential interactions between AFD and AWC, including combinatorial manipulations, would be a valuable direction for future work. In particular, the possibility that AFD exerts a modulatory influence remains an interesting hypothesis to explore.

(3) Given that EAT-4/VGLUT and FLP-6 neuropeptides each contribute to nociception, the authors should consider testing an eat-4; flp-6 double mutant to determine whether this combination of neurochemical signals accounts for AWC signaling to downstream circuits.

We thank the reviewer for this suggestion, which is similar to point 5 of Reviewer 2 (above).

We tested *eat-4*; *flp-6* double mutants and found:

(1) After short-term food deprivation (1hr), *flp-6* and *eat-4* separately contribute to heat evoked reversal at high & low heat and they act in parallel pathways to explain ~90% of animal responsiveness (new version of Fig 3)

Corresponding new text:

“Next, we focused on *eat-4* and *flp-6* mutants, showing the strongest phenotype. We addressed whether glutamate and FLP-6 signaling act dependently of each other in controlling heat-evoked reversal, by testing *eat-4*;*flp-6* double mutants. The residual response seen in each single mutant (Figure 3 A and B) was almost entirely abolished in the double mutant (Figure 3C). A two-way ANOVA for the highest heat stimuli with *eat-4* and *flp-6* genotypes as factors (two levels each: mutant or wild type) showed significant main effects of *eat-4* (*F_(1,67)_* = 48.70, *p*<.001, *η^2^p*=0.421) and *flp-6* (*F_(1,67)_* = 58.50, *p*<.001, *η^2^p*=0.466), respectively, but no interaction effects (*F_(1,67)_* = 0.093, *p*=.761, *η^2^*p=0.001). The significant cumulative effect of the two mutations indicates that the two signaling pathways act mostly independently of each other to mediate heat-evoked reversals.”

(2) After prolong starvation (6hr), *flp-6* mutation has a dominant impact on plasticity and *eat-4* mutation cannot cause loss of plasticity, pointing to a hierarchy in this context (new version of Fig. 6, including revised hierarchy in the model in panel G, and also revised model in Fig. 8).

Corresponding revised text:

“Second, we tested whether starvation-dependent plasticity was preserved in *eat-4* and *flp6* mutant backgrounds, which we had suggested to represent the main AWC transmitters controlling heat-evoked reversals under the early food deprivation condition (Figure 3). Even if the heat-evoked response upon early food deprivation was reduced relative to wild type in *flp-6* mutants, a significant further decline was seen after prolonged starvation (Figure 6B). These results indicate that starvation-dependent plasticity can operate independently of FLP-6. In contrast, *eat-4* mutants displayed markedly elevated heat-evoked responses after prolonged starvation, even exceeding the response level seen in the early food deprivation condition for low heat stimuli (Figure 6C). This potentiated response in *eat-4* mutants was entirely dependent of an intact FLP-6 signaling, since reversal responses in *eat-4*; *flp-6* mutants were entirely abolished, like in *flp-6* single mutant (Figure 6C-E, a two-way ANOVA indicating a significant interaction effect between the two mutations: *F_(1,70)_* = 0.093, *p*<.001, *η^2^p*=0.247). Moreover, the potentiated response in *eat-4* single mutant could not be rescued by expressing *eat-4* rescue transgene selectively in either AWC^OFF^ or AWC^ON^ neurons (Figure 6F). Interestingly, AWC^OFF^-specific rescue produced a further potentiation of heat-evoked reversal response to high heat stimuli (Figure 6F, 6 and 8°C thermal increases), aggravating the phenotype of *eat-4* mutants. These results are consistent with a model in which glutamatergic signaling regulates heat-evoked reversals in starved animals via two bidirectional drives (Figure 6G). On the one hand, glutamatergic signaling—originating from AWC^OFF^—up-regulates reversals in response to high heat stimuli, thus contributing to prevent starvation-induced thermonociceptive plasticity. On the other hand, glutamatergic signaling—originating from neurons other than AWC— down-regulates reversals over a broad range of heat intensities, thus promoting starvation-induced thermonociceptive plasticity. The latter glutamatergic signaling inhibitory effect seems to be more dominant and to depend on intact FLP-6 signaling.”

(4) The authors should consider representing AWC responses to thermal stimuli as histograms to illustrate how fasting increases the probability of some responses and decreases the probability of others.

We thank this reviewer for the suggestion. The proposed histograms nicely convey the concept of “shift in response polarity distribution” that we observed (using the new terminology we now use, instead of using the term “stochastic”). To create such histograms, we computed the magnitude of the peaks on a trial-by-trial basis. When ASI is intact, we see a shift from mostly excitatory responses to an ~equal mix of excitatory and inhibitory responses (new Figure 4C-D). This effect is lost when ASI is ablated (new Figure 5C-D).

(5) It seems important to better understand the ins-6 mutant phenotype and determine whether ASI-to-AWC signaling involves this insulin-like peptide (ILP). The authors should consider using some of the tools available for disrupting ILP signaling to more clearly demonstrate that a specific neurochemical signal mediates modulation of AWCs by ASIs.

Our work identified two ASI-expressed neuropeptides that function to decrease reversal response after starvation: INS-32 and NLP-18. We initially set a lower priority on INS-6 because the reversal response level in starved mutants appeared lower than that in *nlp-18* and *ins-32*. It is important to note that *ins-32* and *nlp-18* are not ‘generally potentiated’ mutants, but display reversal up-regulation selectively following starvation, which placed them as strong candidates to selectively mediate ASI regulation. This said, it is also true that these two mutants (and *ins-6* too) display reduced responsiveness at the early food deprivation time point. Therefore, none of the neuropeptide mutants was strictly identical to ASI, suggesting that the peptides might also work via non-ASI cells at the early food deprivation timepoint (as follow up data indicated at least for *nlp-18*).

Following this reviewer’s comment (and the similar one in the public review), we have attempted to complement our story with the idea of using a similar approach and rescue *ins-6* with its endogenous promoter or ASI-specific promoter. Unfortunately, we failed to obtain rescue effects, and therefore these data remain inconclusive (as we cannot guarantee that the rescue constructs were functional). We decided to keep these data aside in the revised manuscript. Globally, our point made graphically in Figure 7F remains valid. We have complemented the figure legend to mention that INS6 could also potentially work from ASI, but it is not depicted as no ASI-specific data are available. In summary, our data suggests that the connection between ASI and AWC(s) might be established by the integrated action of multiple peptides and their receptors. Further calcium imaging experiments in single, double and potentially triple mutant(s) of peptides and receptors would be required to go deeper in this question, which could be performed in future work.
